# Addition of Lidocaine Injection Immediately before Physiotherapy for Frozen Shoulder: A Randomized Controlled Trial

**DOI:** 10.1371/journal.pone.0118217

**Published:** 2015-02-25

**Authors:** Wei-Chun Hsu, Tao-Liang Wang, Yi-Jia Lin, Lin-Fen Hsieh, Chun-Mei Tsai, Kuang-Hui Huang

**Affiliations:** 1 Graduate Institute of Biomedical Engineering, National Taiwan University of Science and Technology, Taipei, Taiwan, R.O.C; 2 Somategrity Clinic, Taipei, Taiwan, R.O.C; 3 Department of Physical Medicine & Rehabilitation, Shin Kong Wu Ho-Su Memorial Hospital, Taipei, Taiwan, R.O.C; 4 School of Medicine, Fu Jen Catholic University, New Taipei City, Taiwan, R.O.C; 5 National Defense Medical Center, Taipei, Taiwan, R.O.C; 6 University of Taipei, Taipei, Taiwan, R.O.C; Genentech Inc., UNITED STATES

## Abstract

The intraarticular injection of lidocaine immediately before a physiotherapy session may relieve pain during the stretching and mobilization of the affected joint in patients with a frozen shoulder, thus enhancing the treatment effect. To compare the effects of intraarticular injection of lidocaine plus physiotherapy to that of physiotherapy alone in the treatment of a frozen shoulder, a prospective randomized controlled trial was conducted in the rehabilitation department of a private teaching hospital. Patients with a frozen shoulder were randomized into the physiotherapy group or the lidocaine injection plus physiotherapy (INJPT) group. The subjects in the INJPT group underwent injection of 3 ml of 1% lidocaine into the affected shoulder 10 to 20 minutes before each physiotherapy session. In each group, the treatment lasted 3 months. The primary outcome measures were the active and passive range of motion of the affected shoulder. The secondary outcome measures were the results of the Shoulder Disability Questionnaire, the Shoulder Pain and Disability Index, and the 36-item Short-Form Health Survey (SF-36). The outcome measures were evaluated before treatment and 1, 2, 3, 4, and 6 months after the start of treatment. The group comparisons showed significantly greater improvement in the INJPT group, mainly in active and passive shoulder range of motion in flexion and external rotation and improvements in pain and disability (*P* < 0.05); however, no significant group difference was seen in the SF-36 results. The intraarticular injection of lidocaine immediately before a physiotherapy session might be superior to physiotherapy alone in the treatment of a frozen shoulder.

**Trial Registration:**

ClinicalTrials.gov NCT01817348

## Introduction

Frozen shoulder, or adhesive capsulitis of the shoulder, is a common clinical condition characterized by insidious and progressive pain and the loss of active and passive range of motion (ROM) of the glenohumeral joint. The ROM of the shoulder is usually limited to a capsular pattern (mainly external rotation, followed by abduction and flexion; internal rotation is the least commonly involved). In addition, passive movements at or beyond the range limits are painful and resisted movements are not necessary to reproduce or aggravate the pain. The annual incidence of frozen shoulder is 3% to 5% in the general population and as high as 20% in people with diabetes [1]. The causes and mechanism of this syndrome remain unclear [2,3].

A considerable proportion of patients with a frozen shoulder are treated with physiotherapy, nonsteroidal antiinflammatory drugs (NSAIDs), intraarticular injection of corticosteroids, and, recently, intraarticular injection of hyaluronate [4–7]. In persistent cases, more aggressive interventions, such as hydrodilation, manipulation under anesthesia, and arthroscopic or open capsule release, have been performed [8–11].

A variety of physical therapy interventions are used, including superficial application of heat or ice, ultrasound, interferential therapy, transcutaneous electrical nerve stimulation, active and passive ROM exercises, stretching exercises, proprioceptive neuromuscular facilitation techniques, and mobilization techniques [4,5,7,12].

The intraarticular injection of corticosteroids and/or physical therapy programs, including exercise, physical agents such as heat and electrical therapy, and mobilization, are the most common treatment options for patients with a frozen shoulder [12–15]. However, the evidence that shows these options to improve pain and function or alter the natural history of frozen shoulder is controversial [16]. In addition, the side effects of corticosteroids are concerning.

Although therapeutic exercise, particularly stretching exercises and joint mobilization, remains the mainstay of conservative treatment for a frozen shoulder, shoulder pain during the intervention reduces the treatment effect. Manipulation or arthroscopic release under general anesthesia allows the pain experienced during the intervention to be avoided [8,17–19]; however, an increased risk of humeral shaft fractures and failure to release the pathologic tissue have been reported [20]. Moreover, general anesthesia is a major procedure that has inherent risks, is relatively expensive, and may not be accepted by many patients with a frozen shoulder [20, 21].

A practical compromise of intraarticular injection of lidocaine followed by stretching exercises and joint mobilization may be a better method because it avoids the problems related to general anesthesia and allows the patient to be free of pain during the intervention. The purpose of this study was to compare the efficacy of intraarticular injection of lidocaine plus a physiotherapy program (INJPT) to physiotherapy alone (PT) in the treatment of a frozen shoulder. We hypothesized that there would be no significant difference in the efficacy of the INJPT and PT groups in the treatment of a frozen shoulder regarding demographic data and the primary and secondary outcome measures. A rejection of this null hypothesis may help us to examine whether the intraarticular injection of lidocaine immediately before a physiotherapy session could provide pain relief during the subsequent capsule-stretching and joint-mobilization program, thus achieving greater improvement in shoulder pain, ROM, functional ability, and quality of life than does physiotherapy alone.

## Materials and Methods

The protocol for this trial and supporting CONSORT checklist are available as supporting information; see S1 CONSORT Checklist and S1 Protocol.

### Participants

The following inclusion criteria were applied: a) a unilateral frozen shoulder, defined as greater than 50% limitation of passive ROM relative to the nonaffected side in one or more of three movement directions (i.e., abduction in the frontal plane, forward flexion in the sagittal plane, or external rotation in 0° of abduction) with a hard end feel [5,22]; and b) symptoms that had lasted for at least 3 months. The following exclusion criteria were applied: a) previous manipulation of the affected shoulder under anesthesia; b) other rheumatic conditions involving the shoulder (e.g., rheumatoid arthritis, ankylosing spondylitis, or osteoarthritis); c) fracture or dislocation of the affected shoulder; d) previous shoulder surgery; e) Hill-Sachs lesion, severe osteoporosis, or malignancies in the shoulder region); f) neurologic deficits affecting shoulder function; g) disorders of the cervical spine, elbow, wrist, or hand; h) a history of allergy to lidocaine; i) pregnancy or lactation; and j) corticosteroid injection in the affected shoulder during the preceding 4 weeks.

### Design

This prospective and randomized controlled study recruited patients with a frozen shoulder from the outpatient clinic of the Department of Physical Medicine and Rehabilitation at Shin Kong Wu Ho-Su Memorial Hospital. The study project and consent form were approved by the hospital’s ethics committee. From April 2010 through December 2013, we initiated this clinical trial, which was institutional review board–registered from December 2009 to November 2010, and from December 2012 to December 2013. The data reported in this study were collected from patients who were enrolled from January 2013 to December 2013. All of the participants gave written informed consent before they were randomized and baseline measurements were performed. The authors confirm that all ongoing and related trials for this intervention are registered. The patients were randomized to either the PT group or the INJPT group using a table of random numbers. After baseline assessment, sequentially numbered, opaque, sealed envelopes were opened by a research assistant. All injections were administered by a senior physician (L-F, H), who was a board-certified physiatrist and rheumatologist and who had also completed basic and advanced courses in Cyriax’s International Orthopedic Medicine.

### Interventions

In the PT group, each patient underwent electrical therapy and the use of hot packs, followed by stretching exercises and joint mobilization, which were performed by a senior physical therapist three times per week for 3 months or until satisfactory results were achieved. In the INJPT group, lidocaine was injected into the affected shoulder 10 to 20 minutes before the physiotherapy session, but the injection was performed only when the patient suffered from severe shoulder pain (pain score of 7 cm or higher on a 10-cm visual analog scale) during capsule stretching or before posterior joint mobilization. For the injection, the patient was placed in a sitting position with the arm across the abdomen and the elbow flexed to a right angle. The examiner placed an index finger at the coracoid process and a thumb dorsally on the posterior angle where the scapular spine meets the acromion. After sterilization, a 1.5-inch 25-gauge needle was fitted on a 3-ml syringe filled with 3 ml of 1% lidocaine. The same physiotherapy programs were used in both the INJPT and PT groups and were conducted by the same physical therapist. The injections were repeated at a frequency of not more than twice per week and the total number of injections was limited to 10.

### Demographic data

At baseline, data were recorded regarding age, sex, employment status, sports and leisure activities, and history of diabetes mellitus or other medical disorders (items related to the causes of a frozen shoulder), as well as data regarding the duration of complaints, previous treatments, and current pain medications (items related to the stage or severity of the frozen shoulder).

### Shoulder radiography and ultrasonography

To exclude patients with fractures, osteoarthritis, calcification of the tendons, anatomical variants of the acromion, bone tumors, and osteonecrosis, anteroposterior and lateral shoulder radiographs were obtained. We also carried out ultrasound scanning (LOGIQ P5; General Electric Company) of the shoulder, including imaging of the biceps, subscapularis, supraspinatus, and infraspinatus tendons, subdeltoid bursa, and acromioclavicular joint, to rule out rotator cuff tears, tendinopathy, calcification of the tendons, and subdeltoid lesions.

### Outcome measures

The active and passive ROMs of the affected shoulder, including flexion, abduction, internal rotation, and external rotation, were measured with a goniometer by a senior physical therapist who did not know the group assignments of the patients. It has been reported that these goniometric measurements of the shoulder are highly reliable if the measurements are conducted by the same physical therapist [23].

We also used the Shoulder Disability Questionnaire (SDQ) [24] and the Shoulder Pain and Disability Index (SPADI) [24] to quantify the level of pain and disability and the 36-item Short-Form Health Survey (SF-36) to describe general health status. The SDQ includes 16 items developed to evaluate the self-assessed functional limitation of patients with shoulder disorders. The score ranges from 0 to 100, with a higher score indicating a worse condition [25]. The SPADI is a self-administered questionnaire that includes 13 items in two subscales. By averaging the scores from the 5 items of the pain subscale and 8 items of the disability subscale, a score of 0 to 100 is obtained. A high score indicates greater pain or greater disability.

As a general health measurement, the SF-36 comprises eight subscales: physical functioning, role-physical, bodily pain, general health perception, vitality, social functioning, role-emotional, and mental health. A score of 0 to 100 is obtained from each of these subscales, with higher scores indicating better health. From these eight health concepts, two summary scores, one for physical health and one for mental health, can be computed [26].

The patients were evaluated at baseline and at 1, 2, 3, 4, and 6 months after the start of treatment. The deviations of the trial outcomes from the original study protocol were the secondary outcome measures. The Shoulder Rating Questionnaire was replaced by the SPADI, because the SPADI is more commonly used and its validity and reliability are well established. The primary outcome measures were the active and passive ROM of the affected shoulder, and the secondary outcome measures were the scores on the SDQ, SPADI, and SF-36.

### Statistical analysis

An a priori power analysis based on pilot results determined that 27 subjects per group considering the active ROM of flexion and 30 subjects per group considering the active ROM of external rotation would yield a power of 0.8 at a significance level of 0.05. A chi-square test was used to examine the differences in the demographic data between the PT and INJPT groups for categorical variables, including gender, exercise habits, and history of NSAID use. An independent *t* test was used to examine the differences in the demographic data between the PT and INJPT groups for continuous variables, including age, weight, height, and disease duration. The chi-square test was used to test the null hypothesis that there was no significant difference between the PT and INJPT groups in the baseline variables, including gender, exercise habits, and history of NSAID use. The *t* test was used to test the null hypothesis that there was no significant difference between the PT and INJPT groups in the baseline variables, including age, weight, height, and disease duration. For the primary and secondary outcome measurements, the Kolgomorov-Smirnov test was performed to check for normal distribution and homogeneity of variance was checked with Levine’s *F* test. As the assumption of normality was not met for most of the outcome measures, the Mann-Whitney *U* test was used to examine the group effects (i.e., PT and INJPT) and Friedman’s test was used to examine the time effects (evaluation times: before and 1, 2, 3, 4, and 6 months after the start of treatment) on ROM and the SDQ, SPADI, and SF-36 results. All significance levels were set at an α level of 0.05 for comparison of the demographic data in the PT and INJPT groups. Bonferroni adjustments were performed for corrected α levels of 0.025 for the time effects (i.e., calculated for each pairwise comparison in the PT and INJPT groups) and corrected α levels of 0.008 for the group effects (i.e., calculated for six pairwise comparisons for the six evaluation times). SPSS version 19.0 (SPSS Inc., Chicago, USA) was used for all statistical analyses.

## Results

### Baseline characteristics

**Fig 1:**
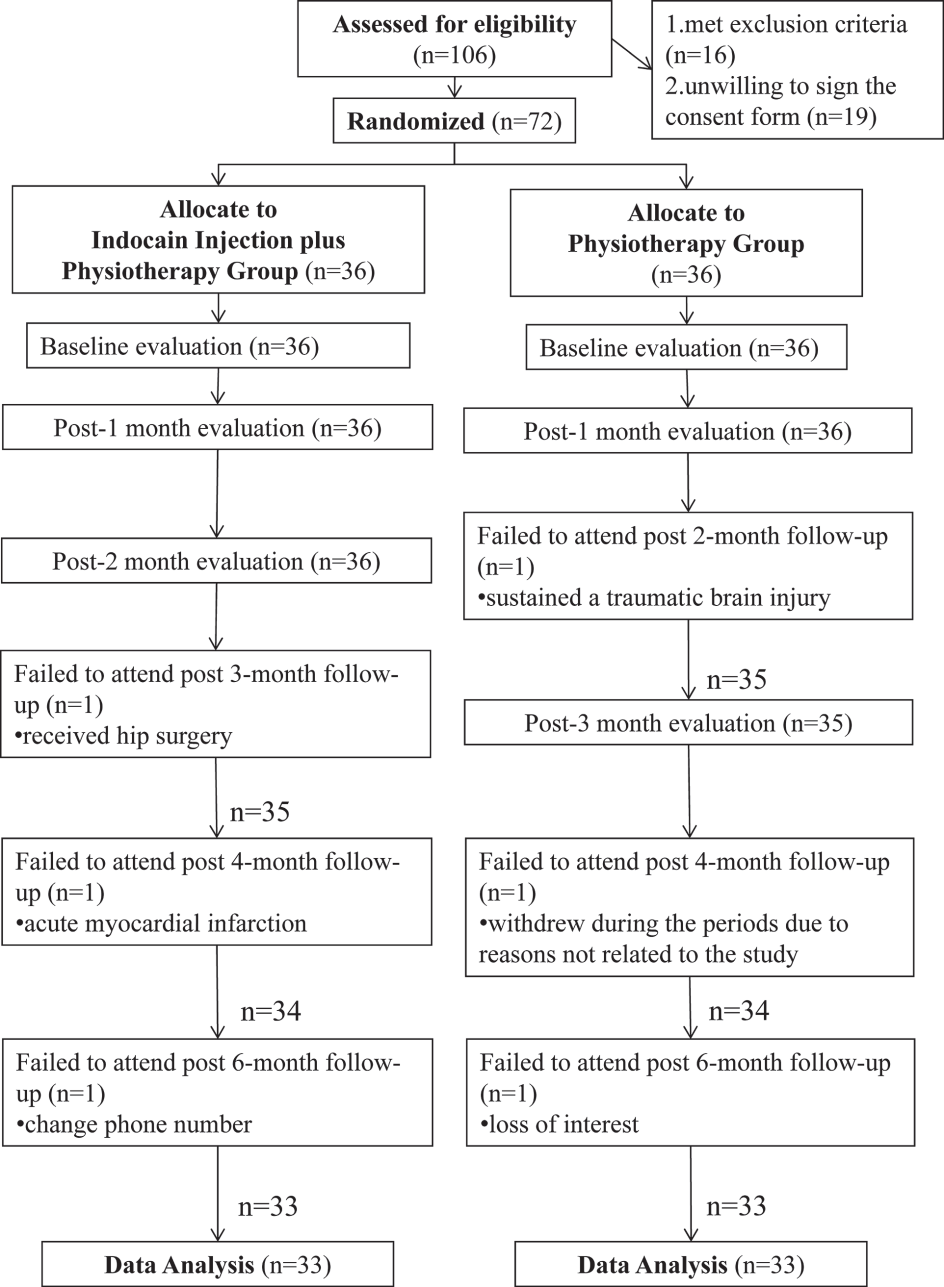
The consolidated standards for reporting trials: a flow diagram of the study. Abbreviations: PT, physiotherapy group; INJPT, lidocaine injection plus physiotherapy group.

We recruited 106 patients for our study. Fifteen were excluded because of the exclusion criteria and 19 refused to sign the consent form. As a result, the study included 36 subjects in the PT group and 36 subjects in the INJPT group (Fig. 1). Of these subjects, three members of each group withdrew during the follow-up period for reasons including traumatic brain injury, acute myocardial infarction, and loss of interest. Thus, 33 subjects in each group completed the study. The baseline characteristics of the participants in the PT and INJPT groups are listed in Table 1; no significant differences in the demographic or baseline measurements were found (Table 1). The average number of injections in the INJPT group was 5.94 ± 2.09 (range, 3 to 10). Mild treatment-related pain in the affected shoulder lasting for 1 to 2 days was reported in two subjects in the INJPT group.

**Table 1:** Demographic and clinical characteristics of the subjects.

Characteristic		PT Group (n = 33)	INJPT Group (n = 33)	
				*P* ^*a*^
Sex	male	8	7	.76
	female	25	26	
Exercise habits	yes	11	13	.60
	no	22	20	
NSAID	yes	17	21	.31
	no	16	12	
				*P* ^*b*^
Age (y)		56.41 ±9.44	54.88 ±7.06	.85
Weight (kg)		62.09 ±9.69	59.61 ± 10.91	.33
Height (cm)		161.09 ±8.37	159.79 ± 7.87	.51
Disease duration (months)		4.54 ±3.25	6.12 ± 5.05	.14

NOTE. Values are expressed as means ± SD or numbers.

Group differences were analyzed using either a chi-squared test or an independent *t* test.

*P* ^*a*^<.05, significant difference from the chi-square

*P* ^*b*^<.05, significant difference from the independent t test

Abbreviations: PT, physical therapy; INJPT, injection plus physical therapy; NSAID, non-steroidal anti-inflammatory drug.

### Range of motion

**Fig 2:**
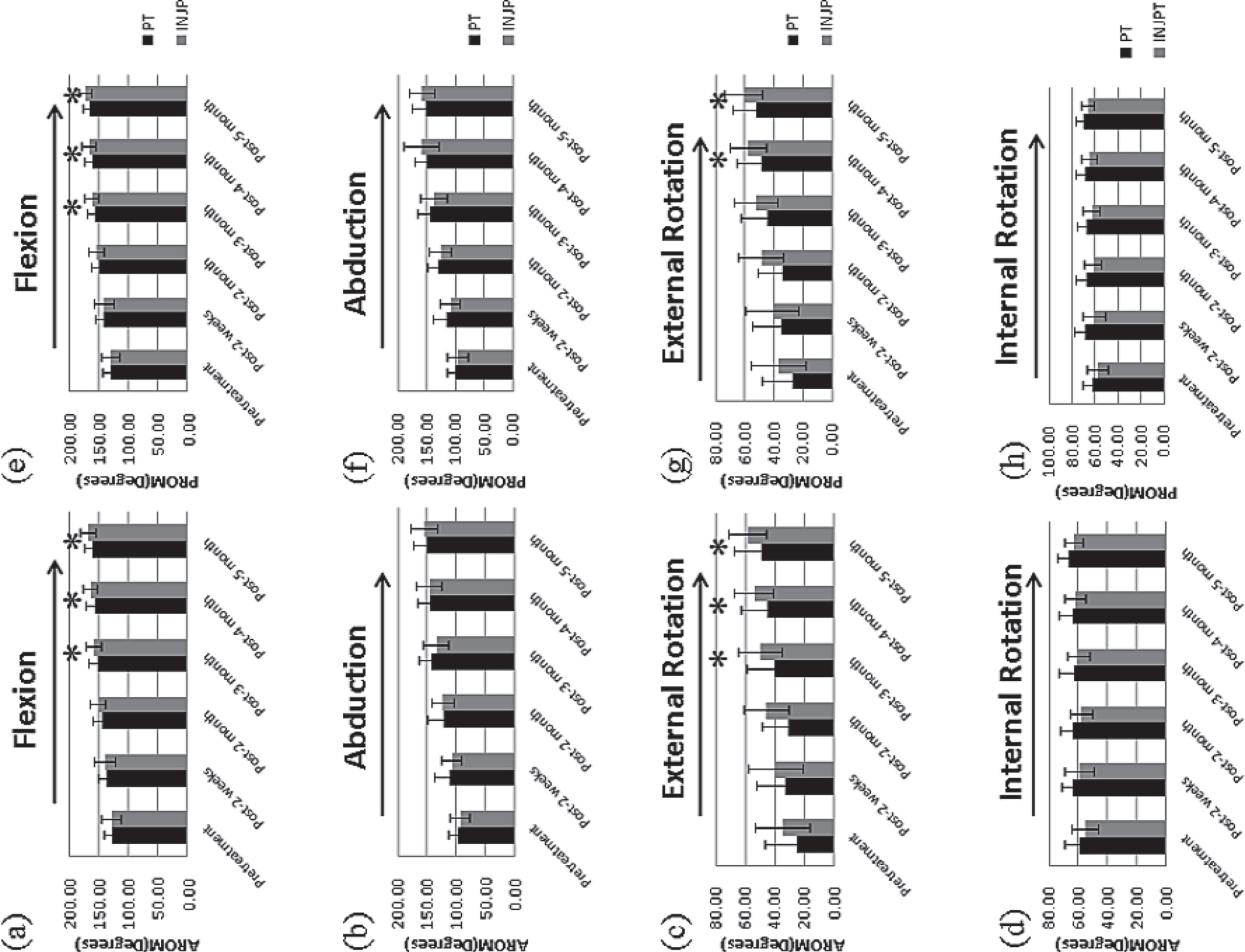
Comparisons of the active and passive ROMs between the groups. Shown as bar charts for (a) flexion, (b) abduction, (c) external rotation, and (d) internal rotation for active ROM and for (e) flexion, (f) abduction, (g) external rotation, and (h) internal rotation for passive ROM with the corresponding standard deviations represented as error bars. An asterisk indicates significant differences between groups (*P*<0.008). For the evaluation times (evaluation times: before and 1, 2, 3, 4, and 6 months after the start of treatment), a right arrow above the graph indicates a significant, linearly increasing trend, whereas a left arrow indicates a significant, linearly decreasing trend (*P*<0.025). (Black bar: the PT group; gray bar: the INJPT group). Group differences were analyzed using Mann-Whitney *U* test. Treatment time effects were analyzed using Friedman's test for two groups respectively. Abbreviations: PT, physical therapy; INJPT, injection plus physical therapy.

In both groups, both active and passive ROM (flexion, abduction, external rotation, and internal rotation) improved over time after treatment (Fig. 2). In the between-group comparison, significant group effects were observed for the active ROM in flexion and in external rotation at 3, 4, and 6 months after treatment. Significant group effects were observed for the passive ROM in flexion at 3, 4, and 6 months after treatment and in external rotation at 4 and 6 months after treatment. Higher values for these active and passive shoulder ROMs were found in the INJPT group than in the PT group (Fig. 2). No significant group effect was observed for internal rotation at any assessment time in either the active or passive ROM.

### Pain and disability

As time passed, the pain and disability after treatment decreased in both groups. For both groups, the pain-related SDQ results (Table 2) indicated that symptoms decreased as time increased after treatment. The SPADI results also indicated that pain, disability, and the total score significantly decreased with time for both groups (Table 2), suggesting that improvements in shoulder-related pain and disability occurred in both groups. In the between-group comparisons, significant group effects favoring the INJPT group were observed for the SDQ at 6 months after treatment and for the SPADI (pain, disability, and total score) at 1 month after treatment (Table 3).

**Table 2:** Effect of time on the secondary outcome measurements.

Scores on Questionnaires		Group	Evaluation Time						Time Effects	
			Pre-treatment	Post-2weeks	Post-2months	Post-3 months	Post-4 months	Post-6 months	Mean Difference of 95% CI.	*P* *
SDQ		PT	48.20 ± 19.03	35.70 ± 18.81	29.28 ± 20.05	29.83 ± 18.96	25.33 ± 18.75	22.61 ± 17.94	0.000 ∼0.146	<. 001*
		INJPT	39.06 ± 7.99	35.03 ± 8.04	27.02 ± 12.4	24.38 ± 14.33	19.86 ± 14.91	10.58 ± 15.72	0.000 ∼0.087	<. 001*
SPADI	Pain	PT	46.36 ± 23.01	32.73 ± 18.66	28.30 ± 17.70	29.82 ± 18.72	24.91 ± 16.56	21.79 ± 16.31	0.000 ∼0.146	<. 001*
		INJPT	55.27 ± 22.43	49.45 ± 27.92	31.03 ± 18.08	25.76 ± 16.69	22.91 ± 18.39	16.73 ± 14.27	0.000 ∼0.087	<. 001*
	Disa	PT	36.25 ± 18.47	28.03 ± 18.50	23.00 ± 17.24	21.98 ± 19.38	18.77 ± 17.42	16.86 ± 14.48	0.000 ∼0.146	<. 001*
		INJPT	54.55 ± 22.47	45.04 ± 22.79	30.34 ± 19.63	26.25 ± 19.78	21.97 ± 19.49	16.74 ± 16.60	0.000 ∼0.087	<. 001*
	Total	PT	41.31 ± 19.68	30.38 ± 17.85	25.65 ± 16.83	25.44 ± 18.94	21.31 ± 15.99	19.32 ± 14.75	0.000 ∼0.146	<. 001*
		INJPT	54.91 ± 20.48	47.25 ± 22.34	30.69 ± 17.93	26.00 ± 17.65	22.44 ± 17.88	16.73 ± 14.81	0.000 ∼0.087	<. 001*
SF-36	Physical functioning	PT	75.61 ± 15.45	77.88 ± 16.25	79.70 ± 13.93	79.70 ± 14.13	81.12 ± 15.18	83.30 ± 13.87	0.000 ∼0.146	<. 001*
		INJPT	71.21±18.79	73.94±19.83	76.67±17.93	76.97±18.03	80.76±18.33	81.67±17.62	0.000 ∼0.087	<. 001*
	Role-physical	PT	44.70 ± 39.80	57.88 ± 40.29	63.67 ± 34.60	64.61 ± 32.10	65.36 ± 32.27	75.33 ± 35.82	0.000 ∼0.146	<.001*
		INJPT	37.88 ± 41.98	46.97 ± 46.25	65.15 ± 42.82	62.88 ± 41.51	71.97 ± 38.91	75.76 ± 39.27	0.000 ∼0.087	<. 001*
	Bodily pain	PT	52.58 ± 13.69	62.27 ± 17.42	66.61 ± 15.97	62.39 ± 16.45	67.33 ± 14.81	70.73 ± 17.91	0.000 ∼0.146	<. 001*
		INJPT	44.55 ± 18.80	55.24 ± 18.46	61.70 ± 15.79	65.85 ± 15.49	66.39 ± 14.55	70.06 ± 17.87	0.000 ∼0.087	<. 001*
	General health	PT	64.18 ± 19.83	58.52 ± 19.70	62.12 ± 18.57	59.91 ± 18.52	62.03 ± 80.82	64.76 ± 18.03	0.000 ∼0.146	<. 001*
		INJPT	58.61 ± 19.85	60.42 ± 20.05	65.39 ± 18.98	64.27 ± 20.10	65.48 ± 19.38	64.76 ± 19.07	0.000 ∼0.142	.006*
	Vitality	PT	63.79 ± 19.29	62.88 ± 17.28	58.94 ± 14.83	60.88 ± 16.10	62.45 ± 16.66	65.97 ± 15.57	0.000 ∼0.146	<. 001*
		INJPT	55.91 ± 21.45	58.18 ± 18.24	59.39 ± 19.91	61.06 ± 20.53	61.52 ± 18.81	61.97 ± 20.42	0.407 ∼0.744	.577
	Social functioning	PT	79.17 ± 19.43	81.06 ± 22.78	80.95 ± 14.34	82.36 ± 12.72	84.77 ± 13.82	87.27 ± 13.02	0.000 ∼0.146	.001*
		INJPT	79.55 ± 19.47	80.68 ± 15.96	81.06 ± 18.25	81.82 ± 14.01	85.23 ± 12.29	84.09 ± 12.99	0.050 ∼0.313	.272
	Role-emotional	PT	74.75 ± 37.30	79.80 ± 35.30	74.14 ± 37.74	77.27 ± 35.38	75.19 ± 35.66	79.75 ± 33.29	0.302 ∼0.751	.474
		INJPT	67.68 ± 42.07	77.78 ± 37.88	76.77 ± 37.72	79.8 ± 38.13	83.84 ± 32.41	86.87 ± 28.79	0.000 ∼0.087	.003*
	Mental health	PT	68.61 ± 19.80	69.33 ± 18.35	66.48 ± 16.65	68.33 ± 17.45	69.97 ± 17.47	71.42 ± 17.17	0.000 ∼0.146	.002*
		INJPT	68.85 ± 20.90	69.33 ± 18.57	67.03 ± 19.47	68.73 ± 18.67	67.88 ± 18.53	64.97 ± 21.05	0.811 ∼1.000	.845

NOTE. Values are expressed as means ± SD or numbers.

*Treatment time effects were analyzed using Friedman's test for two groups respectively.

*P<.025, significant difference

Abbreviations: CI., confidence interval; PT, physical therapy; INJPT, injection plus physical therapy; SDQ, Shoulder Disability Questionnaire; SPADI, Shoulder Pain and Disability Index; Disa, Disability; SF-36, 36-item Short-Form Health Survey

**Table 3:** Effect of group on the secondary outcome measurements.

			PT Group	INJPT Group	Mean Difference of 95% CI.	*P* *
SDQ		Pre-treatment	43.62 ± 17.55	39.06 ± 7.99	0.000 ∼0.096	.116
		Post-1 month	35.70 ± 18.81	35.03 ± 8.04	0.956 ∼1.000	.990
		Post-2 months	29.28 ± 20.05	27.02 ± 12.4	0.395 ∼0.636	.590
		Post-3 months	29.83 ± 18.96	24.38 ± 14.33	0.126 ∼0.328	.192
		Post-4 months	25.33 ± 18.75	19.86 ± 14.91	0.290 ∼0.528	.242
		Post-6 months	22.61 ± 17.94	10.58 ± 15.72	0.000 ∼0.044	. 007*
SPADI	Pain	Pre-treatment	55.27 ± 22.43	46.36 ± 23.01	0.000 ∼0.096	.147
		Post-1 month	49.45 ± 27.92	32.73 ± 18.66	0.000 ∼0.044	.004*
		Post-2 months	31.03 ± 18.08	28.30 ± 17.70	0.504 ∼0.738	.639
		Post-3 months	25.76 ± 16.69	29.82 ± 18.72	0.276 ∼0.512	.379
		Post-4 months	22.91 ± 18.39	24.91 ± 16.56	0.457 ∼0.695	.508
		Post-6 months	16.73 ± 14.27	21.79 ± 16.31	0.126 ∼0.328	.199
	Disa	Pre-treatment	47.35 ± 18.71	36.25 ± 18.47	0.000 ∼0.063	.162
		Post-1 month	45.04 ± 22.79	28.03 ± 18.50	0.000 ∼0.044	.002*
		Post-2 months	30.34 ± 19.63	23.00 ± 17.24	0.065 ∼0.238	.154
		Post-3 months	26.25 ± 19.78	21.98 ± 19.38	0.320 ∼0.559	.415
		Post-4 months	21.97 ± 19.49	18.77 ± 17.42	0.457 ∼0.695	.585
		Post-6 months	16.74 ± 16.60	16.86 ± 14.48	0.457 ∼0.695	.676
		Total	Pre-treatment	49.37 ± 17.35	41.31 ± 19.68	0.000 ∼0.073	.185
		Post-1 month	47.25 ± 22.34	30.38 ± 17.85	0.000 ∼0.044	.002*
		Post-2 months	30.69 ± 17.93	25.65 ± 16.83	0.139 ∼0.346	.256
		Post-3 months	26.00 ± 17.65	25.44 ± 18.94	0.860 ∼0.988	.868
		Post-4 months	22.44 ± 17.88	21.31 ± 15.99	0.840 ∼0.978	.913
		Post-6 months	16.73 ± 14.81	19.32 ± 14.75	0.441 ∼0.680	.473
SF-36	Physical functioning	Pre-treatment	75.61 ± 15.45	71.21±18.79	0.364 ∼0.605	.485
		Post-1 month	77.88 ± 16.25	73.94±19.83	0.349 ∼0.590	.370
		Post-2 months	79.70 ± 13.93	76.67±17.93	0.334 ∼0.575	.469
		Post-3 months	79.70 ± 14.13	76.97±18.03	0.457 ∼0.695	.642
		Post-4 months	81.12 ± 15.18	80.76±18.33	0.689 ∼0.887	.842
		Post-6 months	83.30 ± 13.87	81.67±17.62	0.840 ∼0.978	.954
	Role-physical	Pre-treatment	44.70 ± 39.80	37.88 ± 41.98	0.364 ∼0.605	.502
		Post-1 month	57.88 ± 40.29	46.97 ± 46.25	0.395 ∼0.636	.556
		Post-2 months	63.67 ± 34.60	65.15 ± 42.82	0.165 ∼0.380	.270
		Post-3 months	64.61 ± 32.10	62.88 ± 41.51	0.457 ∼0.695	.547
		Post-4 months	65.36 ± 32.27	71.97 ± 38.91	0.032 ∼0.180	.061
		Post-6 months	75.33 ± 35.82	75.76 ± 39.27	0.065 ∼0.238	.094
	Bodily pain	Pre-treatment	52.58 ± 13.69	44.55 ± 18.80	0.012 ∼0.140	.086
		Post-1 month	62.27 ± 17.42	55.24 ± 18.46	0.022 ∼0.160	.136
		Post-2 months	66.61 ± 15.97	61.70 ± 15.79	0.042 ∼0.200	.139
		Post-3 months	62.39 ± 16.45	65.85 ± 15.49	0.654 ∼0.861	.713
		Post-4 months	67.33 ± 14.81	66.39 ± 14.55	0.441 ∼0.680	.485
		Post-6 months	70.73 ± 17.91	70.06 ± 17.87	0.654 ∼0.861	.661
	General health	Pre-treatment	64.18 ± 19.83	58.61 ± 19.85	0.139 ∼0.346	.264
		Post-1 month	58.52 ± 19.70	60.42 ± 20.05	0.603 ∼0.821	.621
		Post-2 months	62.12 ± 18.57	65.39 ± 18.98	0.457 ∼0.695	.550
		Post-3 months	59.91 ± 18.52	64.27 ± 20.10	0.537 ∼0.766	.608
		Post-4 months	62.03 ± 80.82	65.48 ± 19.38	0.504 ∼0.738	.551
		Post-6 months	64.76 ± 18.03	64.76 ± 19.07	0.840 ∼0.978	.862
	Vitality	Pre-treatment	63.79 ± 19.29	55.91 ± 21.45	0.042 ∼0.200	.164
		Post-1 month	62.88 ± 17.28	58.18 ± 18.24	0.290 ∼0.528	.350
		Post-2 months	58.94 ± 14.83	59.39 ± 19.91	0.956 ∼1.000	.995
		Post-3 months	60.88 ± 16.10	61.06 ± 20.53	0.860 ∼0.988	.954
		Post-4 months	62.45 ± 16.66	61.52 ± 18.81	0.603 ∼0.821	.686
		Post-6 months	65.97 ± 15.57	61.97 ± 20.42	0.234 ∼0.463	.375
	Social functioning	Pre-treatment	79.17 ± 19.43	79.55 ± 19.47	0.840 ∼0.978	.916
		Post-1 month	81.06 ± 22.78	80.68 ± 15.96	0.441 ∼0.680	.500
		Post-2 months	80.95 ± 14.34	81.06 ± 18.25	0.781 ∼0.946	.833
		Post-3 months	82.36 ± 12.72	81.82 ± 14.01	0.620 ∼0.835	.768
		Post-4 months	84.77 ± 13.82	85.23 ± 12.29	0.569 ∼0.794	.637
		Post-6 months	87.27 ± 13.02	84.09 ± 12.99	0.077 ∼0.257	.156
	Role-emotional	Pre-treatment	74.75 ± 37.30	67.68 ± 42.07	0.320 ∼0.559	.516
		Post-1 month	79.80 ± 35.30	77.78 ± 37.88	0.840 ∼0.978	.912
		Post-2 months	74.14 ± 37.74	76.77 ± 37.72	0.504 ∼0.738	.606
		Post-3 months	77.27 ± 35.38	79.8 ± 38.13	0.248 ∼0.480	.376
		Post-4 months	75.19 ± 35.66	83.84 ± 32.41	0.054 ∼0.219	.103
		Post-6 months	79.75 ± 33.29	86.87 ± 28.79	0.126 ∼0.328	.177
	Mental health	Pre-treatment	68.61 ± 19.80	68.85 ± 20.90	0.781 ∼0.946	.842
		Post-1 month	69.33 ± 18.35	69.33 ± 18.57	0.586 ∼0.808	.723
		Post-2 months	66.48 ± 16.65	67.03 ± 19.47	0.882 ∼0.997	.913
		Post-3 months	68.33 ± 17.45	68.73 ± 18.67	0.840 ∼0.978	.933
		Post-4 months	69.97 ± 17.47	67.88 ± 18.53	0.349 ∼0.590	.504
		Post-6 months	71.42 ± 17.17	64.97 ± 21.05	0.139 ∼0.346	.194

NOTE. Values are expressed as means ± SD or numbers.

*Group differences were analyzed using Mann-Whitney U test.

*P<.008, significant difference

Abbreviations: CI., confidence interval; PT, physical therapy; INJPT, injection plus physical therapy; SDQ, Shoulder Disability Questionnaire; SPADI, Shoulder Pain and Disability Index; Disa, Disability; SF-36, 36-item Short-Form Health Survey.

### Quality of life

Significantly increased scores for physical functioning, role-physical, bodily pain, and general health were observed in both groups with increasing time after treatment. There were also significant improvements in vitality, social functioning, and mental health in the PT group, whereas the scores for role-emotional remained statistically unchanged over time in the PT group (Table 2). In the group comparison, no significant group effect was found at any assessment time for these quality of life items (Table 3).

## Discussion

To our knowledge, this randomized controlled trial is the first to confirm the additional benefit of intraarticular injection of lidocaine immediately before a physiotherapy session in the treatment of a frozen shoulder. Although intraarticular injection of corticosteroids or hyaluronic acid has been previously reported for this condition, studies of intraarticular injection of lidocaine to relieve pain during subsequent capsule stretching or joint mobilization have not yet been reported. The combination of intraarticular injection of corticosteroids and physiotherapy in the treatment of a frozen shoulder has been reported, but no temporal relationship between the injection and the physiotherapy session has been established. Other strengths of our study included randomization of the subjects and comprehensive evaluations of pain, functional ability, and quality of life over a relatively long period compared with previous studies [8,13,27].

Neviaser and Hannafin defined four stages of frozen shoulder [7]. Stage 1 is characterized by a gradual onset of pain with no limitations of movement after the pain is relieved with an intraarticular injection of anesthetic. Arthroscopy during this stage reveals a fibrinous inflammatory reaction without adhesion formation. Stage 2 (the freezing stage) is characterized by a combination of synovitis and progressive capsular contracture. Stage 3 (the frozen stage) is the stage of maturation, in which the main patient complaint is significant joint stiffness; a limited ROM is also prominent. Stage 4 (the thawing stage) is the chronic stage; the pain is minimal and gradual improvements in motion can occur [2,7]. All of our study subjects had a limited ROM with a hard end feel and synovitis was either minimal or absent. Although precise clinical classification is difficult, all of our study subjects were in stages 2 to 4; in these three stages, physical modalities followed by stretching and joint mobilization are mostly recommended. However, physiotherapy is not always effective because of the pain experienced during exercise programs [28]. An injection of lidocaine immediately before stretching or mobilization exercises can effectively relieve pain during the exercise programs, thus enhancing the effectiveness of the physiotherapy. Another cause of the enhanced effectiveness might be the capsular stretching effect from the injection of 3 ml of 1% lidocaine into the joint capsule.

In patients with a frozen shoulder, the limitations in the ROM usually follow a capsular pattern, i.e., external rotation is involved earliest and most severely, followed by abduction and flexion, and internal rotation is the least commonly involved. With regard to the primary outcome measurements, our results showed significantly greater improvements in external rotation and flexion in the INJPT group, which indicates an additional effect of the lidocaine injection. With regard to secondary outcome measurements, no group difference was observed in the SF-36, but the INJPT group showed superior results for some items of the SDQ and SPADI. The reason for this difference may be that the SF-36 is a general measurement of quality of life and a change in shoulder ROM may not reflect changes in the SF-36. The SDQ and SPADI were designed for the evaluation of shoulder disorders and are more sensitive to the improvement of shoulder ROM.

Lidocaine hydrochloride, the most widely used local anesthetic, is a reversible blocker of conduction along the small nerve fibers that carry pain and autonomic impulses. The effects occur within seconds and the block lasts for approximately 60 to 90 minutes (for 1% lidocaine) [29]. In our study, stretching or mobilization of the joint was performed 10 to 20 minutes after the intraarticular injection of lidocaine; therefore, the pain experienced during the exercise can be blocked by lidocaine. The use of lidocaine is much safer than that of glucocorticoids, which are associated with many local and systemic side effects, including steroid arthropathy, joint and soft tissue infection, subcutaneous atrophy, skin depigmentation, suppression of the hypothalamic-pituitary axis, and impaired diabetic control [30,31]. Moreover, intraarticular glucocorticoid injection may be effective only in the inflammatory stage of frozen shoulder and the use of corticosteroid injection in the fibrotic stage may be inappropriate.

Over the past few years, chondrotoxicity due to intraarticular injection of local anesthetic has been reported. One clinical report showed that a high-dose infusion of intraarticular local anesthetic (377 ml of 0.5% bupivacaine) by a pain pump was related to chondrotoxicity [32]. Another in vitro study demonstrated a significant decrease in chondrocyte viability after single-dose 1% lidocaine injection [33]. Although chondrotoxicity caused by intraarticular injection of local anesthetics is level IV evidence, to avoid injury to the articular cartilage, we injected the affected shoulder with only 3 ml of 1% lidocaine and kept the number of injections as low as possible. Injections were performed only when passive capsule stretching induced shoulder pain greater than 7 on a 10-cm visual analog pain scale, with a limit of 10 total injections. Two subjects in the INJPT group complained of pain after the physiotherapy sessions, but the symptoms subsided after the intensity of capsule stretching and joint mobilization was decreased. Because of the analgesic effect of the lidocaine, the patients lost the ability to protect their bodies from extrinsic injury. We recommend that manual techniques (following lidocaine injection) should be applied with caution (e.g., guided by the end feel) or by an experienced doctor or physical therapist. No cases of infection or clinical signs of articular cartilage injury occurred in this study throughout the follow-up period.

A variety of physical therapy interventions are currently used to treat frozen shoulder, including the application of heat or ice, ultrasound therapy, interferential therapy, transcutaneous electrical nerve stimulation, active and passive ROM exercise, static stretching or stretching using proprioceptive neuromuscular facilitation techniques, and mobilization techniques [2,5,7,9,34]. Bal et al. [35] concluded that exercise therapy was critically important in the treatment of frozen shoulder. The authors suggested the importance of patient education regarding improvements in shoulder ROM. Stretching should be the focus of treatment, but it should not be performed beyond the limits of the available shoulder ROM. In this study, the immediate pain relief and increased ROM could be explained by the effects of the lidocaine injection. However, the effects disappeared in a few hours, and the long-term effectiveness was mostly a result of physiotherapy.

### Study limitations

The present study has several limitations. First, we did not include an untreated control group for ethical and practical reasons. However, because most of our subjects had had shoulder pain for longer than 3 months without signs of spontaneous improvement, we believed that spontaneous recovery in a short period was unlikely. Second, we were unable to blind the treating physical therapist, so potential bias may have occurred during physical therapy. Third, a placebo effect may have occurred in the injection group. Fourth, a previous study showed that the accuracy of palpation-guided injection of the shoulder ranged from 74% to 91%; injection under ultrasound guidance may increase this accuracy [36]. However, in this study, the pain level during capsule stretching immediately after the injection decreased by more than 50%; thus, accurate injection can be confirmed. Fifth, our sample size may also be one of the study’s limitations. Finally, we did not adopt a more advanced randomization scheme, such as stratified randomization, because it might have required more administrative effort, more clinical visits, and a longer recruitment time than a simple random sample, and this might have increased the difficulty of the whole process of patient recruitment.

## Conclusions

In conclusion, this study demonstrated that intraarticular injection of 3 ml of 1% lidocaine into the affected shoulder immediately before a physiotherapy session can relieve pain during stretching or joint mobilization and thus enhances the treatment effects of physiotherapy for a frozen shoulder. However, in consideration of possible articular cartilage toxicity by local anesthetics, the number of injections should be kept as low as possible.

## Supporting Information

S1 CONSORT Checklist CONSORT Checklist. (DOC)

S1 Protocol Trial Protocol. (PDF)
