# Correction: Addition of Lidocaine Injection Immediately before Physiotherapy for Frozen Shoulder: A Randomized Controlled Trial

**DOI:** 10.1371/journal.pone.0125289

**Published:** 2015-04-15

**Authors:** The PLOS ONE Staff

Fig 1 and Fig 2 are incorrect. Please view correct versions of the figures here.

**Fig 1:**
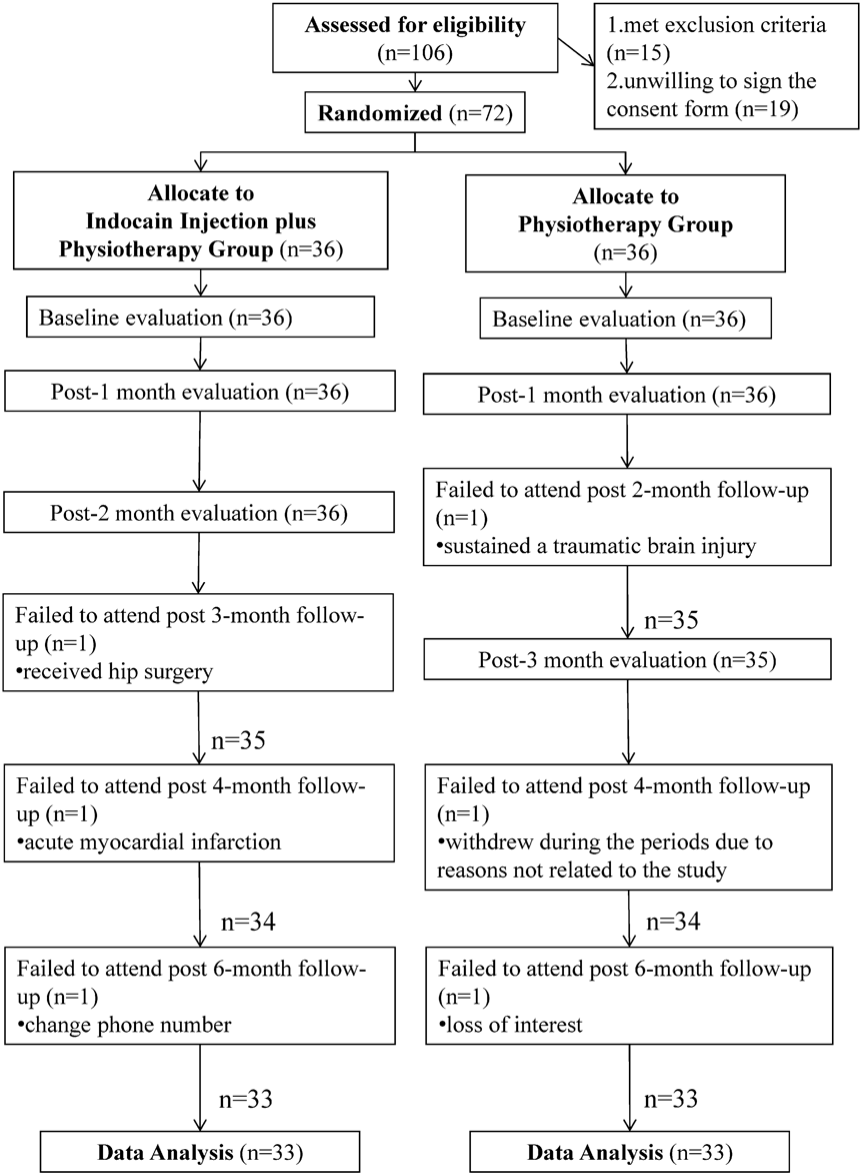
The consolidated standards for reporting trials: a flow diagram of the study. Abbreviations: PT, physiotherapy group; INJPT, lidocaine injection plus physiotherapy group.

**Fig 2:**
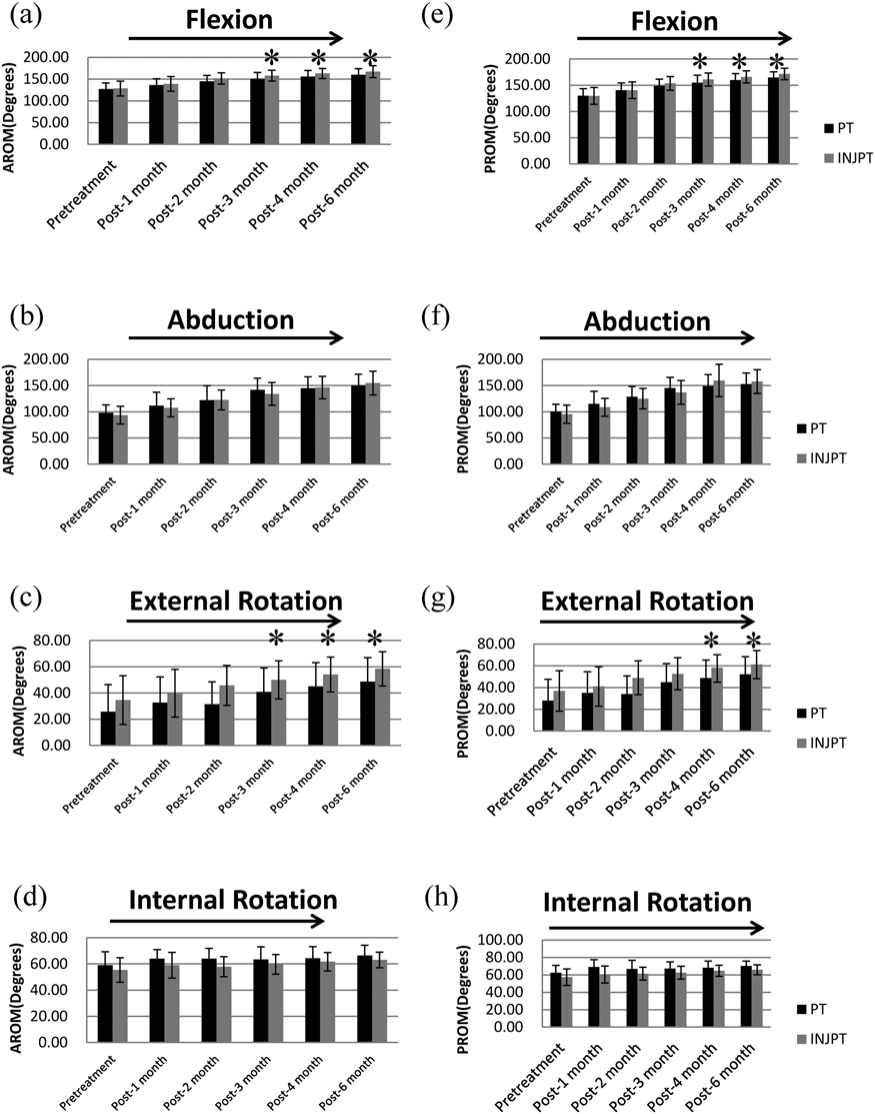
Comparisons of the active and passive ROMs between the groups. Shown as bar charts for (a) flexion, (b) abduction, (c) external rotation, and (d) internal rotation for active ROM and for (e) flexion, (f) abduction, (g) external rotation, and (h) internal rotation for passive ROM with the corresponding standard deviations represented as error bars. An asterisk indicates significant differences between groups (*P*<0.008). For the evaluation times (evaluation times: before and 1, 2, 3, 4, and 6 months after the start of treatment), a right arrow above the graph indicates a significant, linearly increasing trend, whereas a left arrow indicates a significant, linearly decreasing trend (*P*<0.025). (Black bar: the PT group; gray bar: the INJPT group). Group differences were analyzed using Mann-Whitney *U* test. Treatment time effects were analyzed using Friedman's test for two groups respectively. Abbreviations: PT, physical therapy; INJPT, injection plus physical therapy.

There are errors in Table 1 and Table 2 of the published article. Please view the correct tables here.

**Table 1:** Demographic and Clinical Characteristics of the Subjects

Characteristic		PT Group (n = 33)	INJPT Group (n = 33)	
				*P* ^*a*^
Sex	male	8	7	.76
	female	25	26	
Exercise habits	yes	11	13	.60
	no	22	20	
NSAID	yes	17	21	.31
	no	16	12	
				*P* ^*b*^
Age (y)		56.41 ± 9.44	54.88 ± 7.06	.85
Weight (kg)		62.09 ± 9.69	59.61 ± 10.91	.33
Height (cm)		161.09 ± 8.37	159.79 ± 7.87	.51
Disease duration (months)		4.54 ± 3.25	6.12 ± 5.05	.14

NOTE. Values are expressed as means ± SD or numbers.

Group differences were analyzed using either a chi-squared test or an independent *t* test.

*^a^P*<.05, significant difference from the chi-square

*^b^P*<.05, significant difference from the independent *t* test

Abbreviations: PT, physical therapy; INJPT, injection plus physical therapy; NSAID, non-steroidal anti-inflammatory drug.

**Table 2:** Effect of Time on the Secondary Outcome Measurements

Scores on Questionnaires		Group	Evaluation Time							Time Effects
			Pre-treatment	Post-1month	Post-2months	Post-3 months	Post-4 months	Post-6 months	Mean Difference of 95% CI.	P*
SDQ		PT	48.20 ± 19.03	35.70 ± 18.81	29.28 ± 20.05	29.83 ± 18.96	25.33 ± 18.75	22.61 ± 17.94	0.000 ~0.146	<.001*
		INJPT	39.06 ± 7.99	35.03 ± 8.04	27.02 ± 12.4	24.38 ± 14.33	19.86 ± 14.91	10.58 ± 15.72	0.000 ~0.087	<.001*
SPADI	Pain	PT	46.36 ± 23.01	32.73 ± 18.66	28.30 ± 17.70	29.82 ± 18.72	24.91 ± 16.56	21.79 ± 16.31	0.000 ~0.146	<.001*
		INJPT	55.27 ± 22.43	49.45 ± 27.92	31.03 ± 18.08	25.76 ± 16.69	22.91 ± 18.39	16.73 ± 14.27	0.000 ~0.087	<.001*
	Disa	PT	36.25 ± 18.47	28.03 ± 18.50	23.00 ± 17.24	21.98 ± 19.38	18.77 ± 17.42	16.86 ± 14.48	0.000 ~0.146	<.001*
		INJPT	54.55 ± 22.47	45.04 ± 22.79	30.34 ± 19.63	26.25 ± 19.78	21.97 ± 19.49	16.74 ± 16.60	0.000 ~0.087	<.001*
	Total	PT	41.31 ± 19.68	30.38 ± 17.85	25.65 ± 16.83	25.44 ± 18.94	21.31 ± 15.99	19.32 ± 14.75	0.000 ~0.146	<.001*
		INJPT	54.91 ± 20.48	47.25 ± 22.34	30.69 ± 17.93	26.00 ± 17.65	22.44 ± 17.88	16.73 ± 14.81	0.000 ~0.087	<.001*
SF-36	Physical functioning	PT	75.61 ± 15.45	77.88 ± 16.25	79.70 ± 13.93	79.70 ± 14.13	81.12 ± 15.18	83.30 ± 13.87	0.000 ~0.146	<.001*
		INJPT	71.21 ± 18.79	73.94 ± 19.83	76.67 ± 17.93	76.97 ± 18.03	80.76 ± 18.33	81.67 ± 17.62	0.000 ~0.087	<.001*
	Role-physical	PT	44.70 ± 39.80	57.88 ± 40.29	63.67 ± 34.60	64.61 ± 32.10	65.36 ± 32.27	75.33 ± 35.82	0.000 ~0.146	<.001*
		INJPT	37.88 ± 41.98	46.97 ± 46.25	65.15 ± 42.82	62.88 ± 41.51	71.97 ± 38.91	75.76 ± 39.27	0.000 ~0.087	<.001*
	Bodily pain	PT	52.58 ± 13.69	62.27 ± 17.42	66.61 ± 15.97	62.39 ± 16.45	67.33 ± 14.81	70.73 ± 17.91	0.000 ~0.146	<.001*
		INJPT	44.55 ± 18.80	55.24 ± 18.46	61.70 ± 15.79	65.85 ± 15.49	66.39 ± 14.55	70.06 ± 17.87	0.000 ~0.087	<.001*
	General health	PT	64.18 ± 19.83	58.52 ± 19.70	62.12 ± 18.57	59.91 ± 18.52	62.03 ± 80.82	64.76 ± 18.03	0.000 ~0.146	<.001*
		INJPT	58.61 ± 19.85	60.42 ± 20.05	65.39 ± 18.98	64.27 ± 20.10	65.48 ± 19.38	64.76 ± 19.07	0.000 ~0.142	.006*
	Vitality	PT	63.79 ± 19.29	62.88 ± 17.28	58.94 ± 14.83	60.88 ± 16.10	62.45 ± 16.66	65.97 ± 15.57	0.000 ~0.146	<.001*
		INJPT	55.91 ± 21.45	58.18 ± 18.24	59.39 ± 19.91	61.06 ± 20.53	61.52 ± 18.81	61.97 ± 20.42	0.407 ~0.744	.577
	Social functioning	PT	79.17 ± 19.43	81.06 ± 22.78	80.95 ± 14.34	82.36 ± 12.72	84.77 ± 13.82	87.27 ± 13.02	0.000 ~0.146	.001*
		INJPT	79.55 ± 19.47	80.68 ± 15.96	81.06 ± 18.25	81.82 ± 14.01	85.23 ± 12.29	84.09 ± 12.99	0.050 ~0.313	.272
	Role-emotional	PT	74.75 ± 37.30	79.80 ± 35.30	74.14 ± 37.74	77.27 ± 35.38	75.19 ± 35.66	79.75 ± 33.29	0.302 ~0.751	.474
		INJPT	67.68 ± 42.07	77.78 ± 37.88	76.77 ± 37.72	79.8 ± 38.13	83.84 ± 32.41	86.87 ± 28.79	0.000 ~0.087	.003*
	Mental health	PT	68.61 ± 19.80	69.33 ± 18.35	66.48 ± 16.65	68.33 ± 17.45	69.97 ± 17.47	71.42 ± 17.17	0.000 ~0.146	.002*
		INJPT	68.85 ± 20.90	69.33 ± 18.57	67.03 ± 19.47	68.73 ± 18.67	67.88 ± 18.53	64.97 ± 21.05	0.811 ~1.000	.845

NOTE. Values are expressed as means ± SD or numbers.

*Treatment time effects were analyzed using Friedman's test for two groups respectively.

*P<.025, significant difference

Abbreviations: CI., confidence interval; PT, physical therapy; INJPT, injection plus physical therapy; SDQ, Shoulder Disability Questionnaire; SPADI, Shoulder Pain and Disability Index; Disa, Disability; SF-36, 36-item Short-Form Health Survey
