# Supplementary material for: Addition of Lidocaine Injection Immediately before Physiotherapy for Frozen Shoulder: A Randomized Controlled Trial
Source: PLoS One. 2015 Feb 25;10(2):e0118217. doi: 10.1371/journal.pone.0118217 (PMC4340950; doi:10.1371/journal.pone.0118217)
Supplement: S1 Protocol — (PDF) [file pone.0118217.s002.pdf]

# Clinical Trial Protocol Synopsis

Investigator Signature： ___________________

**Protocol title：**

Effect of combined intra-articular injection of lidocain plus physiotherapy in treatment of frozen shoulder

**Objectives：**

The purpose of this study is to compare the efficacy of intra-articular lidocain injection plus physiotherapy and physiotherapy alone in treatment of frozen shoulder.

**Background：**

Although therapeutic exercise (esp. stretch exercise) and joint mobilization remain the mainstay of conservative treatment of frozen shoulder, shoulder pain during the intervention reduce the treatment effect. Manipulation or arthroscopic release during general anesthesia may avoid pain during intervention, however, increased risk of causing a humeral shaft fracture and failure of release of pathologic tissue were reported. Besides, general anesthesia is a major procedure, has inherent risk, is relatively expensive, and may not be accepted by many patients with frozen shoulder.

A compromised way and from a practical point of view, intra-articular injection with lidocain followed by stretch exercise and joint mobilization may be a better method, because it can avoid the problem of general anesthesia and make the patient pain-free during the intervention.

**Study Design：**

Randomized controlled trial: intra-articular injection with lidocaine plus physiotherapy vs physiotherapy alone in treatment of frozen shoulder

**Methods：**

Sixty patients with primary frozen shoulder will be recruited from the outpatient clinic of Department of Physical Medicine and Rehabilitation. The patients will be randomized to 1 of the following 2 treatment groups: group 1, lidocain injection plus physiotherapy (lidocain group); group 2, physiotherapy alone (PT group). Treatments will be continued for 3 months or till the patients gain satisfactory results, but injection with lidocain is performed if pain during intervention equals to or greater than 7-cm in a 10-cm VAS scale, and is limited to 10 times during the whole treatment course. Assessment includes active ROM and passive ROM, measured with a conventional goniometer by a physical therapist; pain will be measured with

three separate visual analog scales; general health status is measured with the 36-Item Shot-Form Health Survey (SF-36); shoulder disability and pain are measured by 2 instruments: the Shoulder Rating Questionnaire (SRQ) and the Shoulder Disability Questionnaire (SDQ). All assessments will be performed at baseline, 1 month, 2 months, and 3 months after beginning of treatment, and 1, 3 months after completion of the treatment. Wilcoxon signed rank test , Mann-Whitney U test , and Student t test will be used for statistics analysis.

**Effect：**

We predict that the effect of intra-articular lidocain injection plus physiotherapy is superior to physiotherapy alone in treatment of frozen shoulder.

**Key words：**

**frozen shoulder, adhesive capsulitis, physiotherapy, lidocain**
